# Providing feedback to frontline paramedics involved in research: a service evaluation within Yorkshire Ambulance Service

**DOI:** 10.29045/14784726.2025.3.9.4.43

**Published:** 2025-03-01

**Authors:** Caitlin Wilson, Fiona Bell

**Affiliations:** Yorkshire Ambulance Service NHS Trust; University of Hertfordshire ORCID iD: https://orcid.org/0000-0002-9854-4289; Yorkshire Ambulance Service NHS Trust ORCID iD: https://orcid.org/0000-0003-4503-1903

**Keywords:** feedback, paramedics, research

## Abstract

**Introduction:**

Many clinical trials within ambulance services require trained frontline paramedics to directly recruit or enrol eligible patients. Research paramedics may then review study documents and often contact recruiting paramedics to provide feedback or obtain missing data. Using the example of the Paramedic Analgesia Comparing Ketamine and MorphiNe in trauma (PACKMaN) study, we aimed to evaluate the provision of feedback by the Yorkshire Ambulance Service (YAS) research team to recruiting paramedics.

**Methods:**

This was a service evaluation using a qualitative design. Data collection was conducted in January 2023 and consisted of a focus group and document analysis. The focus group lasted one hour and involved nine YAS research staff involved in feedback provision. Preliminary results were presented to focus group members to facilitate member checking. Reviewed documents included written feedback sent by the local research team and a standard operating procedure on incidental clinical errors. Data were analysed using the framework method.

**Results:**

Research paramedics provided recruiting paramedics with feedback on ‘research study activities’, such as missing data, (in) eligible patients (not) enrolled or study protocol (not) followed. Feedback on ‘clinical practice’ not related to research activities, but incidentally discovered, was not considered within the remit of research paramedics. Barriers to effective feedback provision were the lack of awareness in recruiting paramedics that feedback would be provided and a perception that unsolicited feedback is mostly negative. Using different formats to provide feedback and engaging in a dialogue with recruiting paramedics was seen to facilitate more meaningful feedback.

**Conclusion:**

Training for recruiting paramedics should highlight that performance and documentation will be scrutinised as part of their involvement in the clinical trial. Although flexibility of local teams regarding feedback format and delivery was important, researchers planning interventional studies in ambulance services may wish to consider the impact of different ways of working with frontline clinicians in their protocol development and study evaluation.

## Introduction

Engaging patients in research has only recently been included in the Health and Care Professions Council’s (HCPC) standards of proficiency for paramedics (HCPC, 2023) and is not always explicitly included in paramedic job descriptions advertised by NHS ambulance trusts. However, many paramedics do choose to be actively involved in research studies, motivated by patient benefit and professional identity (Burges Watson et al., 2012). Active participation in research from a paramedic perspective ranges from completion of surveys or interviews as part of observational studies to recruiting participants to clinical trials.

Due to the ambulance service workforce delivering care in the community in small discrete teams, recruitment of patients to clinical trials often is not carried out directly by dedicated research staff, as would be the case in hospital-based clinical trials, but rather by frontline paramedics. Clinical trials are subject to strict governance, and study protocols must be adhered to (Health Research Authority, 2017). If study protocols are not followed or there are missing data, it is the local research team’s duty to contact the recruiting paramedic to further investigate any potential protocol deviation, seek clarifications or obtain missing data. During this contact the research team report back an element of the paramedic’s performance (for example, ‘Your documentation suggests that you gave this dose of medication’), which could be defined as feedback. Another example of feedback in this context would be the research team contacting a recruiting paramedic to thank them for recruiting a participant.

The provision of feedback to emergency ambulance staff in general is increasingly receiving attention from clinicians, managers and researchers (Cash et al., 2017; Eaton-Williams et al., 2020; Morrison et al., 2017; Wilson et al., 2023a; Wilson et al., 2023b). A recent study conducted interviews with ambulance staff and subsequently developed a typology of pre-hospital feedback (Wilson, Howell, et al., 2022). This typology described pre-hospital feedback from the viewpoint of feedback recipients and did not include feedback provided within the capacity of research studies.

An opinion piece discussing midwifery research notes that regular feedback to clinicians participating in research could promote increased participation (Albers & Sedler, 2004). Given the many barriers faced when encouraging paramedics to participate in research, including the voluntary nature coupled with the additional time research takes (Burges Watson et al., 2012), increasing and maintaining participation is of great interest to researchers in this setting.

Several studies on research delivery and methodology in ambulance services stress the importance of feedback for paramedics in clinical trials. Ankolekar et al. (2014) concluded from the RIGHT trial that paramedics attempting to recruit patients in the challenging pre-hospital care environment should be provided with post hoc feedback. Pocock et al. (2019) found that personal feedback to recruiting paramedics in the PARAMEDIC2 trial fostered engagement and optimised performance. They further described that paramedics wanted to know they had followed procedures correctly in relation both to recruitment to the clinical trial and to their cardiac arrest performance (Pocock et al., 2019).

However, no studies have specifically explored how feedback by research teams to recruiting paramedics is provided or how it could be improved. One recent clinical trial in the ambulance service setting is the Paramedic Analgesia Comparing Ketamine and MorphiNe in trauma (PACKMaN) trial (Michelet et al., 2023). The PACKMaN trial, conducted between 2020 and 2024, compared the effectiveness and safety of ketamine and morphine for pain management in trauma patients treated by paramedics. This clinical trial, funded by the National Institute for Health and Care Research (NIHR), involved the Yorkshire Ambulance Service (YAS) and West Midlands Ambulance Service University NHS Foundation Trust. It aimed to determine whether ketamine provided better pain relief with fewer adverse effects than morphine in the pre-hospital setting.

This service evaluation aimed to assess the provision of feedback by the local research team to paramedics recruiting participants within the PACKMaN clinical trial. The following objectives were addressed:

- Describe the feedback that is provided by the local research team to paramedics recruiting participants within PACKMaN.
- Evaluate whether there have been any changes to the feedback that is provided by the local research team to paramedics recruiting participants within PACKMaN.
- Develop a list of barriers and facilitators for the provision of feedback to paramedics recruiting participants within PACKMaN.
- Generate considerations and tips for best practice regarding the provision of feedback to paramedics recruiting participants within PACKMaN.

## Methods

### Study design

This was a service evaluation using a qualitative design situated within the interpretivist paradigm. According to the Health Research Authority decision-making tool (Health Research Authority, 2022), it was classed as a service evaluation, as it did not seek to produce generalisable or transferable findings. The project was approved and registered as a service evaluation with Yorkshire Ambulance Service NHS Trust.

### Data collection

Work package 1 consisted of a focus group with current and previous staff members of the YAS research department. They were purposively selected based on their direct or indirect involvement in providing feedback to paramedics who were recruiting participants to the PACKMaN trial. An invitation to the focus group was circulated via email to the nine prospective participants, all of whom agreed to participate.

One focus group was held online using Microsoft Teams in January 2023 and had nine participants. The focus group was audio-visually recorded and transcribed using the auto-transcription function in Microsoft Teams. CW then manually checked the transcript for accuracy. CW also made field notes during the focus group. No detailed demographic characteristics of the sample are presented for confidentiality reasons, but the sample encompassed both new and experienced research paramedics.

The focus group lasted one hour and was facilitated by CW, a female senior research fellow paramedic with a PhD in pre-hospital feedback. Sample questions included:

- What feedback was provided?
- How was it provided?
- Who provided the feedback?
- What were the effects of different people providing the feedback?
- Did the trial team provide guidance on what should be fed back to recruiting paramedics? If not, how did you as a team decide what should be fed back?
- Did you consider the ‘lag-time’ of providing feedback?
- What were you trying to achieve by providing feedback?
- Did you consider the ‘timing’ of providing feedback?
- Did you provide only negative or also positive feedback?
- What were the barriers and facilitators?
- Did you ever get feedback on your feedback?

Work package 2 was a document analysis of written feedback sent by the local research team to paramedics recruiting participants within PACKMaN and the standard operating procedure on incidental clinical errors. This was undertaken by CW throughout January and March 2023.

### Data analysis

Data from the focus groups and document analysis were analysed deductively and triangulated by CW using the Framework Method (Gale et al., 2013), supported by the software NVivo (Version 12 Plus, QSR International). Preliminary results were shared with focus-group participants for comments.

Framework analysis (Gale et al., 2013) was chosen for this research study due to its structured approach, which is well suited to addressing the specific objectives outlined. Additionally, its suitability for applied policy research makes it a fitting choice for a study aimed at informing ambulance service research departments and improving participation in research among paramedics. Interview topic guide questions were inspired by a recently developed typology of pre-hospital feedback (Wilson, Howell, et al., 2022), ensuring that the study’s methodology was informed by relevant literature and prior research in the field.

### Reflexivity

Reflexivity in research requires acknowledging personal biases and beliefs, prompting a critical examination of how one’s subjectivity, context, identity and positionality shape the research process (Olmos-Vega et al., 2022; Wilson, Janes, et al., 2022). The authors (FB, CW) are senior members of the YAS Research Institute and were known to the focus group participants. FB had line management responsibilities for the participants and was involved in overseeing the feedback provided within the trial but not actively involved in feedback provision. CW was not involved in line management, feedback provision or oversight but has an interest in feedback for emergency ambulance staff and experience of being a research paramedic for other trials.

## Results

### What feedback is provided?

**Figure 1:**
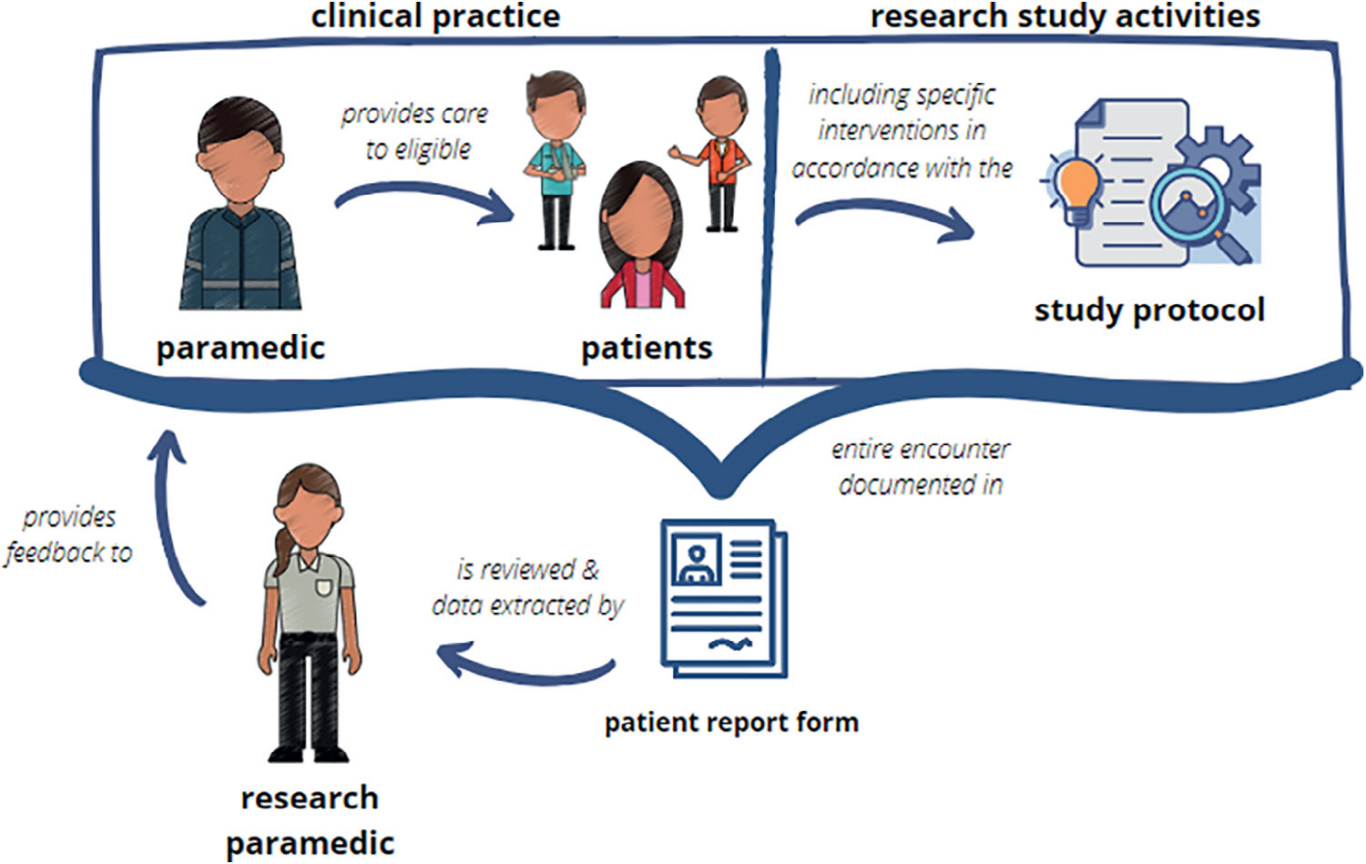
Figure 1. Visual illustration of feedback provided by the research paramedics to recruiting paramedics.

Research paramedics review patient report forms to extract data for the clinical trial. Patient report forms include information on ‘clinical practice’ and ‘research study activities’ of a paramedic–patient encounter. Therefore, there are two potential categories to which ‘feedback’ can relate (see Figure 1):

1. Feedback on ‘research study activities’:
  - Missing data
  - Eligible patients not enrolled
  - Patients incorrectly enrolled as not eligible
  - Study protocol not followed (for example, mistakes, adverse events)
  - Followed all research activities correctly.
2. Feedback on ‘clinical practice’:
  - Patient safety incidents, mistakes or near misses relating to general clinical practice not specifically related to research study activities but discovered by research paramedics when reviewing patient report forms.

### Changes to the feedback provided

In the initial phase, there was no structured process for feedback provision, resulting in feedback being provided on various aspects, including clinical incidents. Typically, feedback was conveyed through conversations with recruiting paramedics. However, this approach posed challenges due to the capacity and clinical expertise of research paramedics. With a high volume of cases and some research paramedics being only recently qualified, correcting errors made by experienced paramedics proved difficult as it was perceived that ‘*clinical incidents are not our [the research paramedics’] domain and should be picked up by team leaders*’ (Participant 6).

The primary aim of research paramedics was to engage paramedics and gather information for the trial database. Over time, it became evident that a significant portion of cases involved ‘*incidental errors*’ (Participant 7). Examples of these included errors in medication dose and contra-indications being overlooked. Consequently, senior organisational stakeholders agreed that research paramedics should only provide feedback on activities directly related to the research study. Clinical practice errors were to be logged using the DATIX system, resulting in the development of the incidental clinical findings process.

Additionally, the research paramedic team established a structured approach to email communication. Template or standard emails were formulated to express gratitude for patient recruitment and to request information pertinent to the study to avoid the perception that research paramedics ‘*were only getting in touch because of a problem and not necessarily getting in touch to say thank you*’ (Participant 2). This standardised communication approach aimed to maintain positive engagement with paramedics involved in the research study by also ‘*feeding back if they haven’t done anything wrong and it’s generally just a “well done” sort of motivation type thing*’ (Participant 3). Participants reflected on the fact that over the course of the study they were sending fewer ‘*you’ve missed this*’ (Participant 8) types of emails and they thought that recruiting paramedics’ documentation was improving, as they ‘*learnt from the initial emails that we sent out*’ (Participant 8).

### Barriers to providing feedback

Barriers to providing feedback within the ambulance service context were identified through various factors.

There existed a prevalent feedback culture within the ambulance service that historically limited feedback provision to critical incidents: ‘*Historically, the only time you every get feedback in operations [is] if you’re going to coroners*’ (Participant 7). Challenges arose from a lack of engagement or negative responses from recruiting staff, with some expressing defensiveness in response to feedback: ‘*getting their back up*’ (Participant 9); ‘*gonna get prickly about it*’ (Participant 6).

The timeliness and format of feedback played a crucial role in moderating these reactions. Participants were conscious of the fact that ‘*emails can be picked up whenever they are able to pick it up but with a phone call they have then got a missed call or it might disturb them when they’re on nights*’ (Participant 4).

Especially at the outset of the study, feedback interactions tended to focus primarily on ‘*reprimanding*’ (Participant 2) paramedics for errors and were ‘*largely seen as providing negative feedback*’ (Participant 2), which could hinder rapport building and constructive dialogue. Communication barriers were compounded by the preference for written communication via email from research paramedics rather than telephone contact. This preference reflected the perceived difficulty in reaching paramedics directly by phone due to patterns of work. Moreover, there was a notable lack of understanding among recruiting paramedics regarding the likely provision of feedback, highlighting the need for clearer communication channels and expectations.

Additionally, research paramedics faced pressure to promptly upload trial data within specified timeframes, necessitating rapid communication with paramedics for further information gathering and feedback purposes. This urgency further strained communication channels and interactions.

### Facilitators to providing feedback

Several facilitators were identified to enhance the process of providing feedback within the context of the study.

The provision of work mobile phones and good access to emails facilitated communication between research paramedics and recruiting staff, ensuring timely and efficient exchange of feedback. Various communication formats, including emails and newsletters, were utilised to disseminate feedback, catering to different preferences and facilitating broader reach among paramedic participants. Furthermore, patient feedback obtained during the consent process was utilised as a valuable source of information – ‘*When I’m consenting patients over the phone, if they’ve got really nice comments to say about the paramedic crew, then I put a GREAT-ix in. It feels a bit less of a token gesture*’ (Participant 6) – and includes direct patient perspectives.

The pre-existing relationship between research paramedics and recruiting staff served as a facilitator, fostering open communication and mutual understanding, thereby enhancing the effectiveness of feedback interactions. Face-to-face feedback sessions were particularly valued, providing an opportunity for in-depth discussions and fostering a more nuanced understanding of perspectives. Such interactions were perceived as more constructive and conducive to relationship building when compared with email correspondence. One research paramedic reflected on a face-to-face interaction resulting in a ‘*really useful discussion about our two perspectives and why I needed some information. It was a lot more in-depth and useful. And she understood where we were coming from, and it just felt a lot nicer than asking for specific information – “you’ve missed this” – over email*’ (Participant 5).

Importantly, paramedics demonstrated a willingness to engage with feedback and expressed a desire to improve their practice, indicating a receptiveness to constructive criticism and professional development.

Some research paramedics had gained experience from other clinical trials and reflected on the fact that certain clinical trials, such as AIRWAYS-2 (Benger et al., 2018), encouraged direct dialogue between researching and recruiting paramedics, facilitating clarification and discussion regarding study procedures and protocols. This direct engagement further strengthened the feedback loop and promoted collaborative learning among paramedic participants.

### Tips for best practice

The following tips were identified as best practices to optimise the feedback process:

1. *Forewarning in training.* It is advisable to inform paramedics during clinical trial training that their documentation will be reviewed by research paramedics, preparing them for feedback, as this may differ from routine clinical practice, where not all documentation is routinely reviewed.
2. *Educational approach to feedback*. Employ an educational approach in providing feedback, carefully choosing the wording (for example, ‘*your documentation suggests*’ (Participant 2)), format and timing of emails. In the medical education literature, Archer (2010) suggests that feedback should ideally be face to face, which would be impractical for most ambulance services, but offering paramedics options for contact methods (such as an informal phone call followed by an email) may be a possible alternative. Providing background information on the reasons for the feedback ensures that the intended goal is clear and can enhance receptiveness (Hattie & Timperley, 2007).
3. *Designated regional research paramedics*. Assigning named research paramedics to each area fosters relationships and opens dialogue channels, enabling recruiting paramedics to provide feedback on research-related aspects. Depending on the nature of the research trial, this also allows research paramedics to offer recruiting paramedics a ‘*pastoral element*’ (Participant 1) by providing a space to ‘*debrief, decompress and often just get stuff off their chest*’ (Participant 1).
4. *Balanced feedback*. Ensure feedback includes both positive and negative aspects, avoiding superficial, irrelevant or insincere communication. ‘*Token gesture emails*’ (Participant 2), ‘*endless chatter*’ (Participant 1) or ‘*throw-away comments*’ (Participant 1) should be avoided. This is consistent with systematic reviews on audit and feedback, suggesting that feedback is most effective when it is specific, timely, individualised and credible (that is, recipients trust in the quality of the data) (Brehaut et al., 2016; Ivers et al., 2012; van der Veer et al., 2010).
5. *Acknowledging variation in practices*. Researchers should acknowledge and accommodate variations in feedback practices across research sites, recognising differences in research and development (R&D) approaches related to local sub-cultures (‘*what they [the local research teams] found works well for them and what they know will be received*’ (Participant 1), ‘*we’re not always going to get it right, but we should know our own staff; we should know the current culture, the political situation, the tone*’ (Participant 1)). Evaluating this variability in trial implementation could offer valuable insights into its impact on results.
6. *Collaboration with operations*. Recognise the need for collaboration with operational teams to address cultural barriers to feedback. Traditionally within ambulance services, feedback has been perceived as linked to disciplinary action or being of questionable value, although more recent studies describe a shift in culture towards being more accepting of discussing mistakes and engaging with feedback (Morrison et al., 2017; Wilson, Howell, et al., 2022). Changing cultural norms may require co-ordinated efforts beyond the research team’s scope (‘*On the cultural stuff – I don’t know whether it will be in the remit of just the research team alone to be able to change that*’ (Participant 2)).

By implementing these best practices, researchers and practitioners can effectively enhance the feedback process, fostering a culture of continuous improvement and collaboration within clinical trial settings.

## Discussion

The service evaluation identified several key findings regarding the reasons for and processes to provide feedback in research in the ambulance setting. Initially, there was no structured process for feeding back to recruiting paramedics, resulting in feedback on various aspects, including clinical incidents. However, over time, it became evident that a significant portion of cases involved ‘incidental clinical errors’, leading to the decision that research paramedics should only provide feedback on activities directly related to the research study. Barriers to providing feedback included a prevalent feedback culture limited to critical incidents, challenges in engagement from recruiting staff and communication barriers. Facilitators included the provision of work phones and good access to emails, pre-existing relationships between research and recruiting staff and face-to-face feedback provision.

### Ambulance service culture

Ambulance culture significantly influences the provision and reception of feedback within this unique clinical environment. Historically, feedback in ambulance services has been predominantly linked to critical incidents, often resulting in perceptions of feedback as punitive rather than constructive (Morrison et al., 2017; Wilson, Howell, et al., 2022). This is compounded by the operational focus of ambulance services, where reflective practices and learning opportunities have often taken a backseat to immediate clinical priorities (McCann, 2022; NHS England, 2024). Within the context of this study, the evolution from informal feedback mechanisms to a more structured approach reflects efforts to navigate these cultural barriers. For instance, the initial challenges of addressing incidental clinical errors, and the potential therein for alienating paramedics, underscore the need for feedback to be framed positively and educationally, as highlighted in the findings.

### Feedback on trial procedures and performance

Our findings support the notion expressed by Ankolekar et al. (2014) that recruiting paramedics should be provided with feedback, and provide guidance on how research teams developing and delivering studies can best provide this.

Similar to Pocock et al.’s (2019) focus-group findings relating to the PARAMEDIC2 study, our findings indicated that feedback to recruiting paramedics could relate to trial procedures and performance. Whereas Pocock et al. (2019) highlighted the desire of recruiting paramedics to receive feedback relating to both trial procedures and performance, our service evaluation suggests that achieving a balance between operational demands and research requirements is essential for fostering a supportive environment conducive to paramedic participation in clinical trials.

### Implications for research and practice

The implications for research and practice underscore a comprehensive approach aimed at optimising feedback mechanisms for paramedics engaged in clinical trials.

First, there is a need to delve into the first-hand experiences of recruiting paramedics regarding feedback within the context of their participation in clinical trials. This exploration would offer valuable insights into their perceptions and requirements regarding feedback reception.

Second, it is crucial to assess various methods of delivering feedback to paramedics involved in clinical trials. By evaluating different options, researchers can identify the most effective and efficient approaches tailored to the needs of paramedic participants.

Third, investigating whether participation in clinical trials serves as a gateway to enhance feedback that paramedics receive directly from patients holds significant importance. Understanding the impact of clinical trial enrolment on feedback mechanisms can potentially improve patient–provider interactions and overall care quality.

Fourth, fostering collaboration between research paramedics and operational team leaders is essential for developing innovative approaches to providing feedback on clinical practice issues. This collaboration could focus on addressing clear errors, such as drug dosages, and on overcoming challenges within clinical trials.

In summary, by adopting a multi-faceted approach encompassing these implications, researchers and practitioners can effectively enhance feedback processes for paramedics involved in clinical trials, ultimately leading to improved patient care and professional development within the paramedic profession.

### Limitations

The service evaluation on feedback to paramedics recruiting into a single study within a single ambulance service has several limitations. First, the potential for bias and subjectivity in the data collected should be acknowledged, particularly in the document analysis of feedback provided to paramedics. Interpretations of the data will have been influenced by the researchers’ perspectives, potentially affecting the validity of the findings.

Second, response bias may have affected the results, as participants in the focus group may have been more inclined to share positive experiences or perceptions of feedback provision. This bias could skew the data and limit the exploration of negative or critical perspectives. Additionally, the study’s lack of longitudinal data may hinder the assessment of changes over time in feedback provision and its impact on paramedics’ practices and attitudes; however, we sought to encompass this by asking research paramedics to reflect on any changes that had occurred.

Other limitations include the study design, which, as a service evaluation, did not seek to establish causal relationships between feedback provision and outcomes such as paramedic drop-out or patient recruitment rates. Finally, the study may not have fully accounted for contextual factors influencing feedback provision, such as organisational culture, workload or resource availability. Addressing these limitations and obtaining recruiting paramedics’ perspectives in future research could enhance the validity and applicability of findings regarding feedback provision to paramedics participating in clinical trials such as PACKMaN.

## Conclusion

The service evaluation on feedback to PACKMaN paramedics revealed variations in feedback provision within the trial, ranging from unplanned informal conversations to templated email communication. Barriers included cultural norms limiting feedback and challenges with communication, while facilitators included access to work phones, pre-existing relationships and paramedics’ receptiveness to feedback as potential barriers.

In conclusion, while flexibility in feedback format is important, researchers planning interventional studies in ambulance services should consider the impact of different feedback implementations in their protocol development. By implementing best practices identified in this study, such as providing forewarning during training and adopting an educational approach to feedback, clinical trial teams can enhance paramedics’ engagement and foster a culture of improvement.

## Acknowledgements

The authors would like to thank the current and previous members of the Yorkshire Ambulance Service Research Institute who attended the focus group and provided feedback on the preliminary findings. Thank you also to the PACKMaN Chief Investigators, Professor Gavin Perkins and Dr Michael Smyth, for supporting the project.

## Author contributions

CW was responsible for study conception and design, data collection and analysis, and drafting and revising the work. FB was responsible for study conception and design, data interpretation and revising the work. CW acts as guarantor for this article.

## Conflict of interest

CW is an associate editor of the BPJ.

## Ethics

Ethical approval was not required for this study, as it was classed as a service evaluation in accordance with HRA guidance. The service evaluation was registered and approved by Yorkshire Ambulance Service NHS Trust (2023-01-06).

## Funding

This service evaluation was not funded. The article received funding from the University of Hertfordshire for open access fees. PACKMaN is funded by the NIHR HTA Programme (NIHR128086). The views expressed herein are those of the author(s) and not necessarily those of the NIHR or the Department of Health and Social Care.
